# Extracting probability in the absence of visual awareness

**DOI:** 10.3758/s13415-022-01057-1

**Published:** 2023-01-26

**Authors:** Shao-Min Hung, Daw-An Wu, Leslie Escobar, Po-Jang Hsieh, Shinsuke Shimojo

**Affiliations:** 1 Faculty of Science and Engineering, Waseda University, Tokyo, Japan; 2 Biology and Biological Engineering, California Institute of Technology, Pasadena, CA USA; 3 Department of Psychology, National Taiwan University, Taipei, Taiwan; 4 Computation and Neural Systems, California Institute of Technology, Pasadena, CA USA

**Keywords:** Uncertainty, Probability, Unconscious processing, Consciousness

## Abstract

Extracting statistical regularities from the environment is crucial for survival. It allows us to learn cues for where and when future events will occur. Can we learn these associations even when the cues are not consciously perceived? Can these unconscious processes integrate information over long periods of time? We show that human visual system can track the probability of location contingency between an unconscious prime and a conscious target over a period of time of minutes. In a series of psychophysical experiments, we adopted an exogenous priming paradigm and manipulated the location contingency between a masked prime and a visible target (i.e., how likely the prime location predicted the target location). The prime’s invisibility was verified both subjectively and objectively. Although the participants were unaware of both the existence of the prime and the prime-target contingency, our results showed that the probability of location contingency was tracked and manifested in the subsequent priming effect. When participants were first entrained into the fully predictive prime-target probability, they exhibited faster responses to the more predictive location. On the contrary, when no contingency existed between the prime and target initially, participants later showed faster responses to the less predictive location. These results were replicated in two more experiments with increased statistical power and a fine-grained delineation of prime awareness. Together, we report that the human visual system is capable of tracking unconscious probability over a period of time, demonstrating how implicit and uncertain regularity guides behavior.

**Supplementary Information:**

The online version contains supplementary material available at 10.3758/s13415-022-01057-1.

## Introduction

The influence of imperceptible external sensory stimuli upon our behavior has generally been regarded as rather automatic (Posner and Snyder, 1975), typically proceeding in a stimulus-driven and attention-free fashion. Although a few studies have shown that an unconscious stimulus can orient bottom-up attention (Hsieh et al., 2011; Hung et al., 2016; Zhaoping, 2008), it is still under debate whether the processing of unconscious information requires top-down attentional resources. Recent studies have begun to show how top-down attention directs unconscious processes. For example, Kiefer and Martens (2010) showed a context-dependent unconscious priming effect where a masked word elicited a stronger semantic effect if it was presented subsequent to a semantic task, as opposed to a perceptual task. That is, how participants’ explicit attention was oriented before the masked prime gated the direction of the implicit priming effect. This finding indicates that the top-down cognitive set can guide unconscious processes, just as it does for conscious processes. More recently, Hung et al. (2020) showed that unconscious semantic interference was dependent on attentional resources. Specifically, a semantic interference between an interocularly suppressed prime and a subsequent visible target only occurred when the task on the target was of low load but not of high load. Together, these findings paint a picture of how attentional control gates unconscious processes, contradicting attention-free accounts of subliminal processing.

Another outstanding question in the field concerns whether unconscious processing is purely stimulus-driven. Is unconscious processing merely driven by the current local stimulus or can information from multiple unconscious stimuli spread across space and time be integrated to form an internal representation? In stark contrast to conscious processing, for which integration has been proposed as a key feature (Dehaene and Changeux, 2011; Oizumi et al., 2014), studies of information processing without consciousness are generally based on individual stimuli, lacking connectivity to the past or future. However, a few studies have recently begun to show that unconscious information can be stored temporarily and utilized subsequently. Soto et al. (2011) showed that a masked orientation could be maintained and used to compare with a target orientation later. That is, the observers showed above-chance performance when asked to discriminate the orientations between an invisible cue and a visible target. Even with an interference from another visible orientation or a 2-second delay, the orientation of the invisible cue still affected performance. In a similar vein, Hung and Hsieh (2021) showed that an interocularly suppressed sequence of words could affect a subsequent lexical decision. The results indicated syntactic integration of two unconscious words presented across several seconds, which could then prime the following visible target word based on their global syntactic congruency. These findings suggest that unconscious information can be held internally for a brief period, which leads to the central question of the current study: Can we integrate information over multiple unconscious stimuli over a lengthened period of time?

**Fig. 1:**
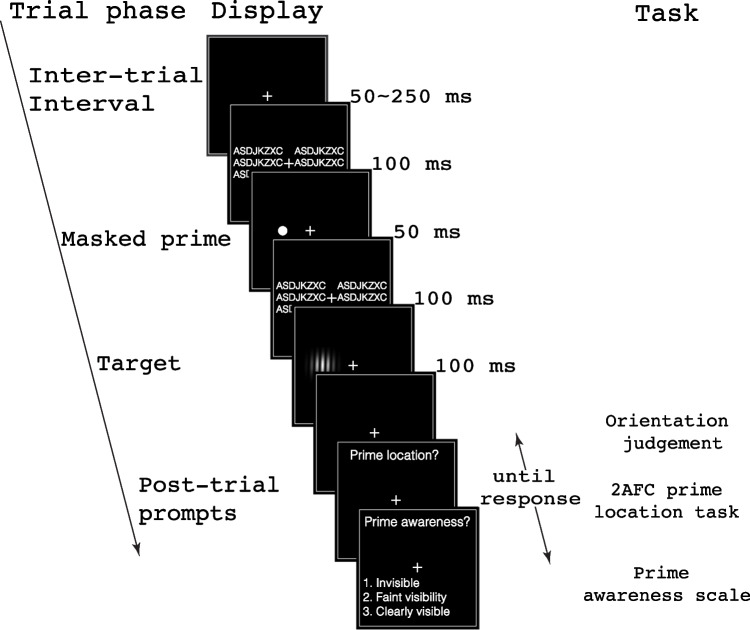
Trial sequence, stimulus, and task. Each trial was preceded by a randomized ITI from 50 to 250 ms. The trial began with a masked location prime. A dot was presented for 50 ms, temporally sandwiched between 100 ms forward and backward masks consisting of meaningless letter strings. These were immediately followed by the 100 ms target, a tilted Gabor patch, on which an orientation judgment was made. After each trial, a 2AFC prime location task was conducted to objectively assess the prime visibility. In Experiments 3 and 4, a prime awareness scale was added to allow the participant to further categorize their subjective awareness of the prime

**Fig. 2:**
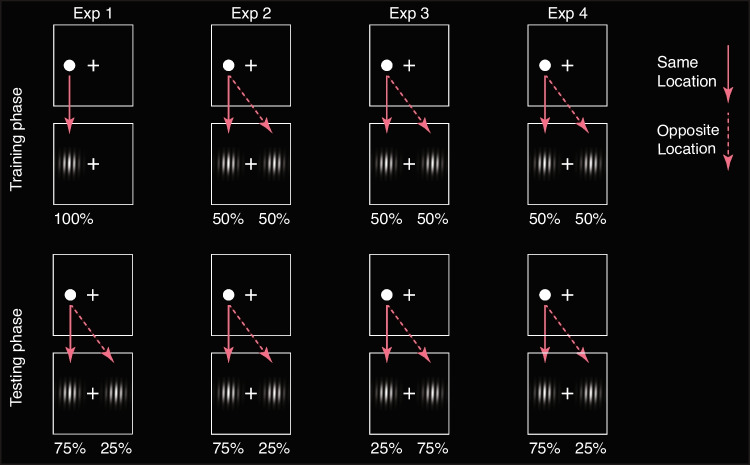
Probability maps for all experiments. Each experiment had a training and a testing phase with distinct prime-target contingencies. This figure illustrates the scenario when the cue appears on the left side

We directly tested whether humans could extract the probability of location contingency between an unconscious prime and a conscious target across dozens of presentations, over a period of several minutes. We asked: Can a change of predictive probability between an unconscious prime and a conscious target, albeit outside one’s visual awareness and knowledge, alter how our visual system takes advantage of the prime? We utilized a spatial cuing paradigm (Fig. 1), with the visual prime sandwiched between a forward and a backward mask. Participants were instructed to judge the orientation of the following target, a slightly tilted Gabor patch. Unbeknown to the participants, the prime-target location contingency (i.e., how well a prime predicted the target location) was manipulated over time (Fig. 2). In the training phase, participants were exposed to an extreme cue-target contingency (either 50% or 100%), whereas in the testing phase, all participants were exposed to an intermediate contingency (75%). If the training phase’s predictive probability between the prime and target could be encoded despite there being no awareness of the prime, we expected to see an altered priming effect in the later trials depending on the contingency training received.

## Methods

### General experimental apparatus

In all experiments, the visual stimuli were generated with MATLAB (The MathWorks, Inc., Natick, MA) and PsychToolbox (Brainard, 1997; Pelli, 1997). Participants viewed the monitor while resting on a chin rest from a distance of 57 cm. The stimuli were presented against a black background on a 21.5-in. iMAC LCD monitor with a resolution of 1,920 × 1,080 pixels and a refresh rate of 60 Hz.

### Participants

All participants (age range: 19–37 years; the average age ranged from 24.0 to 26.8 in different experiments) reported normal or corrected-to-normal vision. They reported no history of psychological disorders and were not on medication at the time of experiment. They gave written, informed consent before the experiment and were reimbursed $5/$10 for participating in a 30-min (Experiments 1-3) or a 45-min (Experiment 4) session. This study was approved by the institutional review board of National Taiwan University and the California Institute of Technology. Participants were excluded before entering the analysis if they 1) had accuracy rates 3 standard deviations away from the group mean on the main task, or/and 2) figured out the experimental design (e.g., correctly reported prime-target relationship in the postexperiment debriefing), 3) failed to achieve desired pre-experiment prime contrast calibration (i.e., chance-rate localization on prime, see Pre-experiment prime thresholding procedure). The excluded number of participants in each experiment was: Experiment 1: *n* = 4; Experiment 2: *n* = 2; Experiment 3: *n* = 4; Experiment 4: *n* = 7. Experiments 1 and 2 aimed at 10 participants based on our pilot experiments (*n* = 9 for both experiments). The sample size in Experiments 3 and 4 was further increased to 20 participants. This decision was based on the power calculation from Experiment 1 data (80% power based on the location congruency effect) and was rounded to the nearest 10. All participants in the final analysis were naïve to the purpose of the experiments.

### Reaction time data pre-analysis processing

All trials that had reaction time shorter than 200 ms were pre-excluded. This number was determined based on the reaction time data in Experiment 1, in which 255 ms was the bottom cutoff of 3 standard deviations away from the average. Reaction times below 200 ms were deemed premature button presses. Furthermore, the reaction time data underwent per-participant per-condition outlier removal to remove data points 2 standard deviations away from the average.

### Pre-experiment prime thresholding procedure

In each experiment, participants first completed a pre-experiment thresholding procedure on prime contrast in which they simply judged the location of the masked prime. This was aimed to acquire individual prime contrast level that led to chance localization of the prime, which served as the frontline of our multiple attempts to ensure the invisibility of the prime. The procedure was identical to that of the main experiment. In each trial, after a varied ITI (intertrial interval) ranging from 50 to 250 ms, a prime disc (0.5°) was presented to the left or right of the fixation point for approximately 50 ms and was sandwiched temporally between two 100-ms noise patterns made of random letters. Participants had to judge the location immediately after the prime presentation. The prime started with 20% of the full alpha value, which was entirely visible, and followed a 1-up-1-down procedure thresholding staircase: if the prime location was correctly reported, the contrast decreased by 3% of the full alpha value in the next trial; otherwise if the location was incorrectly reported, the contrast increased by 3%. The thresholding procedure contained 40 trials, and the final contrast level was taken to the main experiment.

### Experiment 1: Experimental design and procedure

Four blocks, each containing 80 trials, were completed in the main experiment. Unbeknown to the participants, the first two blocks were the training blocks, and the last two blocks the testing blocks. Each trial began with a varied ITI ranging from 50 to 250 ms. Identical to the thresholding procedure, the prime disc (50 ms) was presented to the left or right of the fixation point and temporally sandwiched by two noise patterns (100 ms each). Subsequently, a Gabor patch served as a target and lasted 100 ms. The target was tilted clockwise or counterclockwise for 2 degrees. Participants were asked to first judge the tilt orientation of the Gabor patch as accurately and as quickly as possible and later reported the location of the prime in a two-alternative-force-choice (2AFC) task. Prime location, target location, and target orientation were counterbalanced. The prime-target location congruency was set at 100% (i.e., always on the same location) in the training blocks and at 75% (i.e., on the same location 3 of 4 times) in the testing blocks. To encourage proper fixation, we embedded catch trials in which the participants had to respond promptly when the fixation color changed. These catch trials comprised 20% of all the trials. A trial sequence illustration is provided in Fig. 1.

### Experiment 2: Experimental design and procedure

The design and procedure of Experiment 2 was identical to that of Experiment 1, except that the prime-target location congruency in the training blocks was set at 50% (i.e., the prime had no predictive power of the target). Testing blocks were unchanged from Experiment 1, with 75% contingency.

### Experiment 3: Experimental design and procedure

The design and procedure of Experiment 3 was identical to that of Experiment 2 except for the following changes in the main experiment: 1) The prime-target location contingency was set at 25% in the testing blocks; 2) At the end of the trial, a perceptual awareness scale (Ramsøy and Overgaard, 2004) was added for the participants to report their awareness of the prime with one of the following categories: Not seen at all (Invisible)/Seen but not sure where it was (Faintly visible)/Seen clearly (Visible); 3) The block number of both the training and the testing phases was increased to 3, resulting in a total of 480 trials.

### Experiment 4: Experimental design and procedure

The design and procedure of Experiment 4 were in principle identical to those of Experiment 2 except for the following changes in the main experiment: 1) A perceptual awareness scale was added at the end of each trial to gauge prime visibility; 2) The trial number in each block was increased to 120, resulting in a total of 720 trials; 3) The training phase included the first 2 blocks (identical amount of training trials to that in Experiment 2); 4) Blank trials where no prime was present (while the masks were still presented) were added in the testing phase. These trials served as the baseline to reveal the direction of the congruency effect. The ratio of congruent:blank:incongruent trials was 3:2:1.

## Results

### Confirmation of the unconscious nature of the masked prime in all experiments

All numbers in the results are reported in the format of the mean (*SEM*). In Experiment 1, to establish that the masked prime was objectively invisible, we first calculated the accuracy of the 2AFC prime location task and showed 48.40% (1.65%) (compared with 50%, paired *t*(9) = −0.97, *p* = 0.36). This result indicated successful masking of prime visibility. The result in Experiment 2 did indicate above-chance localization of the prime: 52.11% (0.89%) with paired *t*(9) = 2.37, *p* = 0.04 compared with 50%. We thus further examined the role of prime visibility in the main prime-target congruency effect. The result showed a lack of correlation between the 2AFC location task accuracy and the congruency RT effect (*r* = 0.12, *p* = 0.75), indicating that the priming effect could not be accounted for by prime visibility.

In Experiments 3 and 4, we again first established that the masked prime was objectively invisible, showing the accuracy of the 2AFC prime location task at 50.56% (1.24%) (compared with 50%, paired *t*(19) = 0.45, *p* = 0.66) in Experiment 3 and at 50.38% (1.17%) (compared with 50%, paired *t*(19) = 0.33, *p* = 0.75) in Experiment 4. These results indicated successful masking of prime visibility. Trial-by-trial perceptual awareness scale on the prime was added in both experiments. In Experiment 3, the results showed that a majority (84.45%) of the trials were categorized as “completely invisible,” and only 15% of the trials were categorized as “faintly visible” (i.e., participants felt that they had some faint visibility of the prime but could not reliably localize it) (Fig. S1). In Experiment 4, again a majority (77.72%) of the trials were categorized as “completely invisible,” and 20.16% of the trials were categorized as “faintly visible” (Fig. S1). In Experiments 3 and 4, we included the trials for further analysis that were reported with “completely invisible” or “faintly visible” only when the participant showed chance performance on the 2AFC location task. The chance performance cutoff was applied at 51% accuracy, which was chosen based on the group mean accuracy. If a participant’s accuracy in the faint visibility category exceeded this cutoff, the data were not included.

### Altering probability led to distinct unconscious spatial priming

To ensure proper fixation, we embedded catch trials (20% of total trials) in which participants were instructed to respond to fixation cross color change as quickly and as accurately as possible once it occurred. Because all catch trials had to be responded to in order to proceed, which led to 100% accuracy, we used the percentage of trials not exceeding 2 standard deviations of mean catch trial RT as a proxy of participants’ sustained attention on the fixation cross. In Experiment 1, we found consistent performance in these catch trials: 97.97% (0.24%) of the trials were within the 2 STD threshold. A similar result was acquired in Experiment 2: 97.03% (0.36%).

To examine whether prime-target location congruency affected target response, we directly compared the accuracy (ACC) and reaction time (RT) when the prime and target were presented in the same location (congruent, CON) versus different locations (incongruent, INCON). Before these main experiments, we conducted two pilot experiments with fixed contingency between the prime and target throughout the experiment to first establish the validity of this priming paradigm. With 100% and 50% prime-target location contingency, both showed faster response times in the prime-congruent location (100%: CON: 735.3 ms (29.1 ms) vs. blank 746.7 ms (30.0 ms), *t*(8) = −2.80, *p* = 0.02, Cohen’s d_av_ = 0.39; 50%: CON: 729.0 ms (27.2 ms) vs. INCON 744.6 ms (27.7 ms), *t*(9) = −6.12, *p* = 0.0003, Cohen’s d_av_ = 0.57). However, ACC did not differ (100%: CON: 84.64% (4.14%) vs. INCON 84.20% (3.64%), *t*(8) = 0.50, *p* = 0.63, Cohen’s d_av_ = 0.11; 50%: CON: 90.45% (1.81%) vs. INCON 92.01% (2.07%), *t*(8) = −1.73, *p* = 0.12, Cohen’s d_av_ = 0.80).

**Fig. 3:**
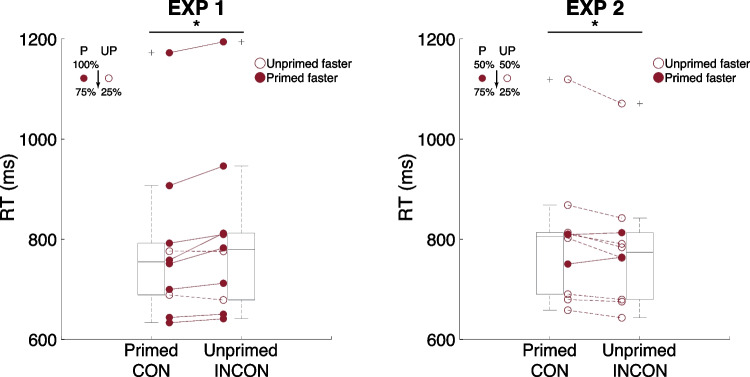
Experiments 1 and 2, testing phase results. The y axis denotes reaction time (RT), and the x axis denotes target location with respect to the prime. Each pair of connected dots represents one participant. Filled dots denote participants with shorter RT in the primed location (CON), whereas open dots denote those with shorter RT in the unprimed location (INCON). A box plot is embedded in the background to show data distribution. Following the convention, the line inside the box denotes median, and the upper and lower limits denote 75th and 25th percentiles. Whiskers extend to 1.5 interquartile range. Crosses represent data exceeding this range. P, primed location; UP, unprimed location. *p < 0.05

The same analyses were performed on the data of the testing phase in Experiments 1 and 2 where the testing phase (75% contingency) occurred subsequent to distinct training phases (Experiment 1: 100% contingency; Experiment 2: 50% contingency). Both experiments showed a priming effect from the unconscious prime, but in opposite directions. In Experiment 1, a paired *t* test showed shorter RT in the congruent trials (CON: 782.4 ms (50.1 ms) vs. INCON 800.4 ms (52.5 ms), *t*(9) = −2.97, *p* = 0.02, Cohen’s d_av_ = 0.35). In Experiment 2, an identical analysis showed shorter RT in the incongruent trials (CON: 800.2 ms (41.6 ms) vs. INCON 782.8 ms (38.0 ms), *t*(9) = 2.88, *p* = 0.02, Cohen’s d_av_ = 0.44) (Fig. 3). Similar to our pilot experiments, ACC did not show a difference between the two conditions (Experiment 1: CON: 89.37% (2.55%) vs. INCON 91.56% (3.46%), *t*(9) = −1.02, *p* = 0.34, Cohen’s d_av_ = 0.73; Experiment 2: CON: 86.15% (2.01%) vs. INCON 88.12% (2.37%), *t*(9) = −1.10, *p* = 0.30, Cohen’s d_av_ = 0.9).

These results showed that a brief training (i.e., 80 trials x 2 blocks, approximately 10 minutes) on the implicit contingency between an unconscious prime and a conscious target induced distinct priming effects. When the prime target was highly contingent in the training, participants later showed a primed location benefit: shorter RT to the primed location. In contrast, when the prime-target was not contingent in the training, shorter RT to the unprimed, low-contingency location was observed. The divergence of priming effects subsequent to distinct implicit prime-target contingency indicated a strong top-down contingency-tracking mechanism that overturned bottom-up, saliency-based, unconscious priming. A direct comparison between the RT difference of the location congruent and incongruent trials in Experiments 1 and 2 supported this reversed pattern (one sample t: *t*(9) = −3.37, *p* = 0.008, Cohen’s d_av_ = 1.95). By top-down, we did not mean that such a mechanism was intentional and willful. Rather, we referred to a learned attention mechanism that was endogenous and clearly against a typical bottom-up saliency map (Itti and Koch, 2001).

It seemed odd that the noncontingency training led to a reversal in the priming effect during the test period, rather than simply weakening or nullifying the priming as one would have expected. In Experiments 3 and 4, we clarified the nature of this reversal, and explored the time course of its development. These further experiments also served to replicate and solidify the reversal result, in case it was merely a chance occurrence, or other artifact. To this end, we doubled the number of participants and increased the trials both in the training and testing periods. Now each experiment was equipped with six blocks (80 trials/block x 3 training blocks in Experiment 3; 120 trials/block x 2 training blocks in Experiment 4). Furthermore, we added a perceptual awareness scale on a trial-by-trial basis for us to better delineate participants’ subjective awareness of the prime (Fig. 1; more details in Methods).

### Low contingency attracted attention subsequent to no contingency training

There are two potential explanations to the results in Experiment 2 where participants responded faster to the unprimed location in the testing phase. After being unconsciously trained with no contingency between the prime and target locations, participants may have been implicitly allocating their attention to the low-contingency location (contingency-based attentional effect). On the other hand, participants may have been simply orienting their attention away from the prime or to the opposite location of the prime (prime-based attentional effect). Experiment 3 was designed to tease apart these two possibilities. We reversed the contingency between the prime and the target in the testing phase so that the prime was only 25% contingent on the target location. Other changes were made to increase the power and allow participants to classify their prime awareness (see Methods for details).

**Fig. 4:**
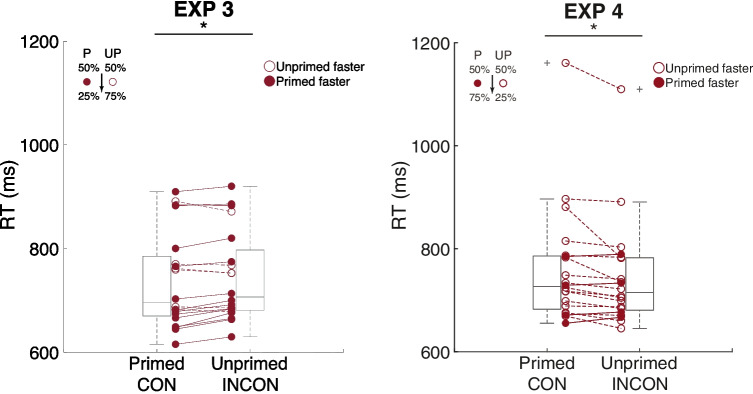
Experiments 3 and 4, testing phase results. The y-axis denotes reaction time (RT), and the x-axis denotes target location with respect to the prime. Each pair of connected dots represents one participant. Filled dots denote participants with shorter RT in the primed location (CON), whereas open dots denote those with shorter RT in the unprimed location (INCON). A box plot is embedded in the background to show data distribution. Following the convention, the line inside the box denotes median, and the upper and lower limits denote 75th and 25th percentiles. Whiskers extend to 1.5 interquartile range. Crosses represent data exceeding this range. P, primed location; UP, unprimed location. *p = 0.01

RT analysis sorted by prime-target location congruency showed faster RT in the congruent trials (25%) with CON: 737.9 ms (20.8 ms) vs. INCON 745.2 ms (19.5 ms), *t*(19) = −2.71, *p* = 0.01, Cohen’s d_av_ = 0.36 (Fig. 4, Exp. 3). This result was consistent with Experiment 2 and showed that attention allocation was drawn toward the *low-contingency location*, rather than away from the prime. Similarly, ACC did not show a difference between the two conditions (CON: 89.36% (2.25%) vs. INCON 89.71% (2.29%), *t*(19) = −0.48, *p* = 0.63, Cohen’s d_av_ = 0.15).

### Further replication of the low-contingency effect and examination of time course

Experiments 2 and 3 showed that after immersing in the noncontingent, unconscious prime-target relationship, subsequent low-contingency oriented participants’ attention. It was unlikely that such an attentional effect was simply based on bottom-up prime saliency. For example, in Experiment 2, participants responded faster to the location opposite to the prime location, indicating that the attention distribution could be top-down and internally driven. Because this was the most critical result in our study, Experiment 4 was designed to directly replicate it in yet another new group of participants. Experiment 4 had all test enhancements that we made in Experiment 3, including doubled number of participants, increased trial number, and inclusion of the perceptual awareness scale in every trial. Furthermore, we added blank trials in the testing period where no prime was presented as the baseline condition to allow assessment of the congruency effect direction.

RT analysis sorted by prime-target location congruency revealed similar results from Experiment 2: Decreased RT in the incongruent trials (25%) with CON: 760.8 ms (26.0 ms) vs. INCON 744.9 ms (23.4 ms), *t*(19) = 2.82, *p* = 0.01, Cohen’s d_av_ = 0.64 (Fig. 4, Exp. 4). Although the mean RT of the blank trials (752.0 ms (21.2 ms)) fell between that of CON and INCON trials, the difference did not reach significance (CON vs. blank *t*(19) = 1.30, *p* = 0.21, Cohen’s d_av_ = 0.42; INCON vs. blank, *t*(19) = −1.50, *p* = 0.15, Cohen’s d_av_ = 0.32). Similar to prior experiments, ACC did not show a difference between the two critical conditions (CON: 89.62% (1.43%) vs. INCON 90.71% (1.45%), *t*(19) = −1.10, *p* = 0.29, Cohen’s d_av_ = 0.76). Nor did ACC show a difference between congruent/incongruent and blank trials (CON vs. blank 89.28% (1.71%), *t*(19) = 0.3, *p* = 0.77, Cohen’s d_av_ = 0.22; INCON vs. blank, *t*(19) = 1.28, *p* = 0.22, Cohen’s d_av_ = 0.91). Experiment 4 thus successfully replicated the results of Experiment 2, with more participants, a larger number of trials, and resulting in an increased statistical significance and effect size.

**Fig. 5:**
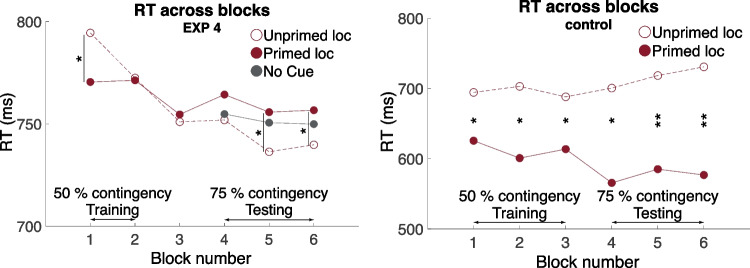
Temporal dynamics of the priming effect in Experiment 4 (LEFT) and a control experiment with conscious primes (RIGHT). **LEFT.** The typical location-based congruency priming was found in the first training block and decreased over time. Distinctively, the decreased RT in the unprimed, low-contingency location was found in the later blocks. **RIGHT.** When the prime was conscious, a typical location-based priming effect was found throughout the experiment. That is, faster RT to the primed location. The effect was stronger when the contingency was higher in the later testing phase. *p < 0.05; **p < 0.01

To assess the temporal dynamics of the priming effect, we further conducted a two-way repeated measures analysis of variance on the RT with prime-target congruency and block number as the factors. A marginal main effect of block was found, *F*(1, 19) = 2.18, *p* = 0.06, η_p_^2^ = 0.1. Furthermore, there was an marginal interaction between congruency and block, F(1, 19) = 2.14, *p* = 0.07, η_p_^2^ = 0.1. Post-hoc comparisons showed the priming effects only in the first block of the training phase (*t*(19) = −2.07, *p* = 0.05) and in the final two blocks of the testing phase (*t*(19) = 2.80, *p* = 0.01 and *t*(19) = 2.35, *p* = 0.03). Critically, the initial priming effect, attributable to the default exogenous cuing, was reversed across the training and testing phases (Fig. 5), reflecting a gradual process that acted to nullify the exogenous priming effect during the training phase, and then acted further during the testing phase, ultimately leading to a fully reversed priming effect in the last two blocks.

### Conscious prime exhibited distinct priming effects and temporal trajectory

We have attempted to ensure that the prime was properly masked and remained invisible in all our experiments (see details in Confirmation of the unconscious nature of the masked prime in all experiments), including 1) showing chance performance in the 2AFC prime location task in most experiments, 2) selecting participants who reported being unaware of the existence of the prime, and 3) adding in trial-by-trial perceptual awareness scale to allow fine-grained categorization of prime experience by each participant, which helped select the trials that both subjective and objective invisibility were established for further analysis. We understand that no single approach could ensure the (in)visibility of a perceptual object, however, these multiple measures served as our best approximation and were meant to be as conservative as possible in ensuring that the prime was invisible.

To further ensure that prime invisibility played a central role in the priming effects reported in these experiments, in a separate control experiment, we asked if conscious primes could exhibit a similar priming effect in our paradigm. That is, if the answer were positive, our results from the unconscious primes could at least partially be explained by a proportion of undetected conscious primes. To this end, we ran a conscious control in which the experimental design and procedure were almost identical to those of Experiment 2 except that all the primes were *not* masked and clearly visible.

Similar analyses were performed. RT analysis sorted by prime-target location congruency revealed the classic location-based priming effect: Decreased RT in the congruent trials (75%) with CON: 575.2 ms (22.5ms) versus INCON 715.5 ms (38.0 ms), *t*(4) = −4.94, *p* = 0.008, Cohen’s d_av_ = 4.64. These results exhibit a strong opposite priming pattern than that driven by unconscious primes, suggesting that it was unlikely that our key results in Experiments 2, 3, and 4 resulted from conscious priming. Furthermore, we looked at the temporal trajectory of the priming effect, which again showed a distinct pattern to that reported in Experiment 4 (Fig. 5). An identical two-way repeated measures analysis of variance on the RT was performed. A main effect of congruence was found, *F*(1, 4) = 20.80, *p* = 0.01, η_p_^2^ = 0.84. Furthermore, there was an interaction between prime location congruency and block, *F*(1, 4) = 3.38, *p* = 0.02, η_p_^2^ = 0.46. Post-hoc comparisons showed the congruency priming effect in all blocks (all *p* < 0.05), whereas stronger effects were observed in the later, higher probability testing blocks (all *p* < 0.03). These results further lent support to the distinctions between conscious and unconscious priming effects in the current paradigm.

## General discussion

We used a series of psychophysical experiments to show unconscious probability tracking in the human visual system. An unconscious location priming effect was guided by the prime-target predictive probability that participants were exposed to over a period of time in the training lasting approximately 10 minutes. A direct comparison of the testing phases in Experiments 1 and 2 showed that the pattern of spatial attention triggered by the prime was dependent on the prime-target contingency contained in the training period. These findings revealed the capacity of our visual system to integrate unconscious information over multiple incidences and a period of time on the order of minutes. Critically, an internal contingency map, which was not entirely driven by external salient sensory stimulations, needed to be formed and updated throughout the experiment to track the frequency of co-occurrences between the prime and the target, supporting a dynamic and flexible unconscious system.

In addition, the particular pattern of priming that emerged was interesting. When the prime effectively predicted target location during the training, participants in subsequent trials responded faster to the primed location. In contrast, when the prime was not predictive of the target location, a faster reaction time was found in the low-contingency location. Two additional experiments further replicated the results of Experiment 2 with increased statistical power, again revealing faster reaction times in the low-contingency location after noncontingency training. The temporal profile of this shift toward the low-contingency location suggested a slow process spanning the whole experiment, where the attention allocation triggered by the prime slowly shifted to the location with low cue-target contingency (Fig. 5. LEFT.).

Furthermore, a control experiment with unmasked primes ruled out the possibility that this unconscious priming was potentially contaminated by a handful of conscious primes. That is, the results in the control experiment clearly showed a classic, stimulus-driven priming with conscious primes. The temporal trajectory of the conscious priming effect was also in stark contrast with that of the unconscious one (Fig. 5). These results indicated the similarities and dissimilarities between conscious and unconscious processes. Both processes responded to the temporal changes in the accumulative statistics of the stimuli. However, the critical feature that oriented attention in the two systems was clearly distinct. The conscious system exhibited an intuitive bottom-up response pattern to the prime (e.g., a saliency map, Itti and Koch, 2001), which was later strengthened by increased predictive probability between the prime and target. Conversely, the unconscious system gave more weight to the rare events and eventually displayed a reversed attention distribution pattern, which resulted in faster access to the low-contingency unprimed location. These findings suggest that a different framework to account for the computation of attention in the unconscious system is needed.

It has been proposed that our perceptual system is predictive and accumulates statistics among sensory items to update our ever-changing representations (Friston, 2005; Heeger, 2017). Recently, a surge of neuroscientific research on how predictive information shapes neural representation has been observed (Ekman et al., 2017; Kok et al., 2012, 2017; Noonan et al., 2016; van Moorselaar and Slagter, 2019). Most studies have reported prestimulus neural tunning for a predictable target (Kok et al., 2017; Noonan et al., 2016; van Moorselaar and Slagter, 2019). Specifically, once a target is expected, neural signals dedicated to the expected feature or location can be picked up before the presentation of the stimulus and extend beyond the target existence. How about a distractor? In a recent study, van Moorselaar and Slagter (2019) showed that behaviorally, the observer benefited from expecting the appearance of a distractor due to distractor location repetition. However, the pre- and post-spatial tuning to distractor location was absent. Furthermore, expecting a distractor also reduced distractor-evoked neural response. These findings suggest that the neural computations of expected targets and distractors are very likely distinct. Coming back to our study, could it be that after the noncontingency training, an unconscious prime was deemed a distractor, because it was not informative? Future research is required to look into scenarios where the predictive values are generated implicitly. Because a direct subjective report of an unconscious distractor is improbable, a better understanding of the underlying neural mechanism could give hints to how an unconscious prime is utilized in the brain.

The results in the current study can be considered in the context of the typical location-priming paradigm (Posner and Snyder, 1975), in which a preceding prime orients the observer’s attention in a bottom-up, exogenous manner. Past studies have consistently documented such a priming effect with conscious cues (Posner et al., 1980, see review by Egeth and Yantis, 1997) or unconscious cues (McCormick, 1997, see review by Mulckhuyse and Theeuwes, 2010). However, simple stimulus-based saliency or exogenous attention capture can explain neither the essential nor the curious aspects of our results.

First, our results showed that unconscious priming could be independent of or even against mere stimulus saliency (e.g., the more salient spatial location) in a manner dependent on the cue-target contingencies present in the training period. The behavioral divergence between Experiments 1 and 2 thus requires an endogenous factor, which is formed as a reaction to the integrated statistical properties of temporally scattered subliminal cues.

Second, and curiously, training with no prime-target contingency led to a situation where, with an increased physical contrast in the primed location that was supposed to attract attention, participants’ attention was not only nulled, but in fact deployed to the low-contingency location. It is worth noting that the no-contingency training does not simply cause attention to be deployed away from subsequent primes but away from subsequent high-contingency locations. Thus, in Experiment 3, reactions times were faster in the primed location, where targets were less frequent. The temporal data from Experiment 4 (Fig. 5) provides more detail as to how this reversal pattern forms. There is a gradual learning process whereby the initial separation in reaction times (attributable to an exogenous priming effect) becomes nulled during the training with no-contingency primes. This nulled state continues for some time into the test period, even though a contingency has been introduced. With sufficient exposure to the new contingency, the priming effect shifts to the low-contingency location. The reversal of the total priming effect indicates that a weakening of exogenous priming would not suffice as an explanation. Instead, there appears to be a build-up of a countervailing endogenous priming process. This is consistent with the descriptive aspects of the time-course data, in which the reaction time to the primed (exogenous) location remains fairly flat, whereas the reaction time to the unprimed location falls, not only in the training phase, but also into the testing phase. A full accounting of this additional learning that leads to reversal is beyond the scope of this study. But this clearly reinforces the idea that the statistics of the subliminal cues are continually being integrated and that the endogenous processing continues to be updated as the statistical landscape changes. Further research is needed to clarify what those processes are. In the meantime, it may be worthwhile to entertain some speculative accounts and connections to similarly shaped findings to bring to light some interesting possibilities.

One possible form of learning that may have occurred during the testing phase to this attraction to low-contingency locations is that after exposure to unpredictable primes in training, the rare events (i.e., the low-contingency targets) in testing became salient and captured attention. To grasp the idea of scarcity, a continuous tracking of prime-target contingency is required. Critically, the priming effect in the testing phase required time to build up and only occurred in the later blocks, indicating an accumulation of prime-target statistics.

The unconscious contingency learning observed in the current study likely resulted from implicit learning rather than explicit strategic learning. Given the verified objective and subjective prime invisibility, participants were oblivious both to the existence of the prime and the prime-target relationship, with the latter confirmed by all participants in the debriefing. At least one prior study has examined whether a seemingly automatic response was triggered by reflexive response, implicit learning, or planned strategy utilizing a go/no-go paradigm (Bissett and Logan, 2012). In a canonical go/no-go paradigm, participants exhibit post-stop-signal slowing, reflecting a typical conflict between executing a go response and suppressing a response upon recently receiving the no-go signal. Bissett and Logan (2012) showed that post-stop-signal slowing was not due to reflexive response but instead could be driven by implicit learning or a shift in strategy. One particular result revealed strong implicit learning: when participants were oblivious to the contingency between the current no-go trial and the subsequent one, they nevertheless exhibited post-stop-signal slowing when the current no-go trial had a higher contingency of preceding another no-go trial (i.e., two consecutive no-go trials). This phenomenon based on implicitly accumulating the contingency of no-go trials was observed in the later stage of the experiment, suggesting gradual learning. These results showed that the participants did *implicitly* acquire some temporal contingency and could utilize the newly learned statistics to guide their behavior. Our study provided an even more “unconscious” scenario where the contingency of the prime and the subsequent target was not only unbeknown to the participants, but the prime also was subliminal, which revealed a novel unconscious contingency learning over time.

It is worth pointing out that our results do not endorse a full-on flexible unconscious system that can adaptively select, maintain, and suppress information based on goal-directed behavior. It is apparent that the participants in the current study were still passively receiving and updating the probability between an unconscious prime and a conscious target. No active and deliberate top-down choices were made to deploy their attention accordingly. In fact, it is the lack of intention and awareness of the prime that made our findings particularly intriguing. The fact that our data show probability tracking over many trials without the observers 1) seeing the prime and 2) knowing the prime-target relationship informs how well our visual system extracts statistical regularities from the environment.

The limited bandwidth of conscious vision has been repeatedly shown (Cohen et al., 2016), with multiple bottlenecks, such as attention and photoreceptor density. If an ability fundamental to our life, such as integrating statistical regularities in our environment, is limited to conscious vision, it is hard to conceive how the massive amount of information can be dealt with moment by moment. We provide a possible solution: unconscious integration of probability over multiple incidences. Our findings thus provide novel evidence to support an active machinery where co-occurrences of individual visual items were unconsciously tracked over a period of time, revealing the adaptive values of our unconscious visual system.

## Supplementary Information

ESM 1 (DOCX 25 kb)
