# Supplementary material for: Extracting probability in the absence of visual awareness
Source: Cogn Affect Behav Neurosci. 2023 Jan 26;23(3):620–30. doi: 10.3758/s13415-022-01057-1 (PMC10390606; doi:10.3758/s13415-022-01057-1)
Supplement: Supplementary file 1 — (DOCX 25 kb) [file 13415_2022_1057_MOESM1_ESM.docx]

**Supplementary materials**

**Extracting probability in the absence of visual awareness**

Shao-Min Hung^1,2*^, Daw-An Wu^2^, Leslie Escobar^2^, Po-Jang Hsieh^3^, Shinsuke Shimojo^2,4^

^1^Faculty of Science and Engineering, Waseda University, Tokyo, Japan

^2^Biology and Biological Engineering, California Institute of Technology, Pasadena, CA, USA

^3^Department of Psychology, National Taiwan University, Taipei, Taiwan

^4^Computation and Neural Systems, California Institute of Technology, Pasadena, CA, USA

*Correspondence: [smhung@aoni.waseda.jp](mailto:smhung@aoni.waseda.jp)

Keywords: uncertainty; probability; unconscious processing; consciousness

1. Fig. S1. Experiments 3 and 4 prime awareness scale report and prime location task performance.

**Fig. S1. Experiments 3 and 4 prime awareness scale report and prime location task performance.** The y axis denotes the percentage. In each figure, the left panel shows the percentage of trials reported in unseen, faintly visible, and seen categories. The right panel shows the performance on the 2AFC location judgment. 50% is the chance.
